# The *Toxoplasma gondii* ME-49 strain upregulates levels of A20 that inhibit NF-*κ*B activation and promotes apoptosis in human leukaemia T-cell lines

**DOI:** 10.1186/s13071-018-2837-1

**Published:** 2018-05-18

**Authors:** Qian Chen, Min-Hui Pang, Xiao-Hong Ye, Guang Yang, Chen Lin

**Affiliations:** 1 Department of Microbiology and Immunology, Medical College, Jinan University, Guangzhou, Guangdong Province 510632 People’s Republic of China; 2 Department of Epidemiology and Health statistics, Medical College, Jinan University, Guangzhou, Guangdong Province 510632 People’s Republic of China; 3 Department of Parasitology, Medical College, Jinan University, Guangzhou, Guangdong Province 510632 People’s Republic of China

**Keywords:** *Toxoplasma gondii* ME-49 strain, A20, ABIN1, Human leukaemia T-cells, Apoptosis

## Abstract

**Background:**

Acute T-lymphocyte leukaemia is a form of haematological malignancy with abnormal activation of NF-κB pathway, which results in high expression of A20 and ABIN1, which constitute a negative feedback mechanism for the regulation of NF-κB activation. Clinical studies have found that acute T-lymphocyte leukaemia patients are susceptible to *Toxoplasma gondii* infection; however, the effect of *T. gondii* on the proliferation and apoptosis of human leukaemia T-cells remains unclear. Here, we used the *T. gondii* ME-49 strain to infect human leukaemia T-cell lines Jurkat and Molt-4, to explore the effect of *T. gondii* on proliferation and apoptosis, which is mediated by NF-κB in human leukaemia T-cells.

**Methods:**

The Tunel assay was used to detect cell apoptosis. Cell Counting Kit-8 was used to detect cell proliferation viability. The apoptosis level and the expression level of NF-κB related proteins in human leukaemia T-cells were detected by flow cytometry and Western blotting.

**Results:**

Western blotting analyses revealed that the *T. gondii* ME-49 strain increased the expression of A20 and decreased both ABIN1 expression and NF-κB p65 phosphorylation. By constructing a lentiviral-mediated shRNA to knockdown the A20 gene in Jurkat T-cells and Molt-4 T-cells, the apoptosis levels of the two cell lines decreased after *T. gondii* ME-49 infection, and levels of NF-κB p65 phosphorylation and ABIN1 were higher than in the non-konckdown group. After knockingdown ABIN1 gene expression by constructing the lentiviral-mediated shRNA and transfecting the recombinant expression plasmid containing the ABIN1 gene into two cell lines, apoptosis levels and cleaved caspase-8 expression increased or decreased in response to T. gondii ME-49 infection, respectively.

**Conclusions:**

Our data suggest that ABIN1 protects human leukaemia T-cells by allowing them to resist the apoptosis induced by *T. gondii* ME-49 and that the *T. gondii* ME-49 strain induces the apoptosis of human leukaemia T-cells via A20-mediated downregulation of ABIN1 expression.

## Background

*Toxoplasma gondii* is an intracellular parasite that can inhibit the proliferation of host cells and induce their apoptosis [1–3]. The immune response to *T. gondii* results in the killing by T-cells or phagocytosis by phagocytic cells [4]. However, as *T. gondii* enters the incubation period, T-cells also exhibit inactivation and even apoptosis, which severely disrupts the normal immune function of the organism [5]. Additionally, during the period of acute infection, host cells often undergo obvious apoptosis, but during the period of chronic infection, only a small number of apoptotic cells have been observed [5, 6]. Therefore, the initiation and development of cell apoptosis may play an essential role in the pathogenesis of toxoplasmosis.

At present, *T. gondii* can induce the apoptosis of host cells *via* the endoplasmic reticulum (ER), death receptors (extrinsic pathway), and the mitochondrial pathway (internal pathway). The ER pathway increases oxidative stress, which is caused by virulence factor ROP18 in *T. gondii* to enhance the expressions of cleaved caspase-12, CHOP and cleaved caspase-3 in the neural cells, which then induce apoptosis via a variety of signaling pathways [7]. The death receptor pathway predominantly increases the expression level of TNFR1 on the cell surface and induces apoptosis by forming death-inducing signalling complex (DISC) to activate downstream caspase-8. Dincel et al. [8] found that the levels of TNFR1 and caspase-8 in the brain significantly increased after *T. gondii* ME-49 infection, and the levels of apoptosis-related proteins in the internal pathways, such as caspase-3 and caspase-9, were significantly upregulated. Mitochondrial pathway mediated apoptosis occurs with the increased release of cytochrome *c* and activation of the downstream caspase-9 kinase. Studies have shown that the infection of trophoblast cells with *T. gondii* leads to structural damage and dysfunction in the mitochondrion, and the downstream caspase-9 and caspase-3 kinase are also significantly activated, finally leading to apoptosis in trophoblast cells. In mesenchymal stem cells, *T. gondii* can induce apoptosis by downregulating the mitochondrial Mcl-1 protein level, Mcl-1 protein strongly interacted with Beclin-1 in the mitochondrion, which decreases LC3B and cleaved caspase-3 levels [9, 10].

In vitro*, T. gondii* may inhibit the proliferation of tumour cells and induce apoptosis, which may be related to the excessive activation of the associated signalling pathway in tumour cells. Clinical studies have found that acute T-lymphocyte leukaemia patients usually have severe immunosuppression and are prone to opportunistic infections with *T. gondii*, which destroys the immune balance of the organism and induces apoptosis of tumour cells [11, 12]. Acute T-lymphocyte leukaemia is a kind of haematological malignancy with abnormal activation of NF-κB pathway, which results in unlimited proliferation of tumour cells [13, 14].

The nuclear factor kappa B (NF-κB) family contains nuclear transcription factors that regulate many of the early-response genes involved in cell proliferation, cell apoptosis, and inflammatory response, and is constitutively active in human cancer cells [15]. Studies have shown that *T. gondii* can affect the proliferation of host cells *via* the NF-κB signalling pathway. Gazzinelli et al. [16] found that the soluble secretory protein of *T. gondii* can activate NF-κB transcription factors in mouse macrophages in vivo; however, little is known about the mechanism of action. Caamano et al. [17] found that the apoptosis level of macrophages increases significantly after NF-κB knockout mice are infected with *T. gondii*, which causes the T-cells to fail to sustain a long immune response. However, in vitro, Shapira [18] found that the invasion of macrophages by *T. gondii* does not lead to the activation of NF-κB, and *T. gondii* significantly decreased the ability of LPS to activate NF-κB. These studies suggest that *T. gondii* has different effects on NF-κB activation in vivo and in vitro, but the effect of *T. gondii* on NF-κB after infection in human leukaemia T-cells in vitro remains unclear.

A20, which has been widely studied, is a protease that performs ubiquitin chain hydrolysis that inhibits NF-κB activation through a negative feedback mechanism. Srivastav et al. [19] found that Protozoa can upregulate the expression of A20 in lymphocytes and evade the immune response of host cells by inhibiting the expression of NF-κB-related pro-inflammatory genes. Kumar et al. [20] found that the expression of A20 protein in lymphocytes is significantly upregulated after mice are infected with *Mycobacterium tuberculosis*, and that in A20-deficient macrophages, the levels of TNF-α, IL-1 and other inflammatory factors are upregulated. These studies show that pathogens can upregulate the expression of A20 in lymphocytes, which can weaken the immune response mediated by NF-κB in responding to pathogenic microbial infections. A20 cannot promote apoptosis, but it indirectly promotes apoptosis by inhibiting the downstream anti-apoptotic gene levels of NF-κB [21–23]. ABIN1 is a newly discovered anti-apoptotic protein that has high expression in lymphocytes and can also help A20 attenuate NF-κB activation [24–27]. Therefore, we hypothesized that *T. gondii* affected NF-κB activation and apoptosis levels by regulating the expression of A20 and ABIN1 in human leukaemia T-cells.

## Methods

### Cell preparation, reagents and antibodies

The *T. gondii* ME-49 strain was provided by the Laboratory of Parasitology, Jinan University. The Jurkat T-cells and Molt-4 T-cells were obtained from the American Type Culture Collection (ATCC). The rabbit monoclonal anti-human antibodies pNF-κBp65, A20, ABIN1, and Cleaved caspase-8 were from Cell Signalling Technology (Boston, USA), and the NF-κB p65, MALT1 antibody was from Abcam (Cambridge, UK). The mouse monoclonal anti-human GAPDH and secondary antibodies were purchased from Santa Cruz Biotechnology (Dallas, USA). shRNA for A20 and ABIN1 were designed and synthesised by Guangzhou OBIO Co, Ltd. (Guangzhou, China). CCK-8 kit was purchased from Dojindo (Kumamoto-ken, Japan). Nuclear and Cytoplasmic Protein Extraction Kit and Annexin V-FITC and propidium iodide (PI) kit were purchased from KeyGen Biotech (Nanjing, China). The TNIP1-3Flag-IRES2-EGFP recombinant plasmid was constructed by OBIO Co., Ltd. (Guangzhou, China). The MALT1 inhibitor MI-2 was purchased from MedChem Express (USA). The TUNEL Cell Apoptosis Detection Kit was purchased from Beyotime Biotechnology (Shanghai, China).

### Cell lines, *T. gondii* ME-49, and culture

The Jurkat T-cells and Molt-4 T-cells were cultured in Roswell Park Memorial Institute (RPMI1640) medium supplemented with 10% fetal bovine serum (FBS, complete medium, Gibco) at 37 °C in a humidified atmosphere of 5% CO_2_. The *T. gondii* ME-49 strain was cultured in human foreskin fibroblasts (HFF cells) at 37 °C in Dulbecco's modified eagle medium (DMEM) with 10% FBS.

### Purification of *T. gondii* ME-49

Tachyzoites of *T. gondii* must be purified before infection with T-cells. After the HFF cells at the bottom of the cell bottle were scraped with a cell scraper, the cell suspension was pipetted with a 5 ml syringe, and blown out by replacing the needle with a needle for a 1 ml syringe. Finally, the tachyzoites were purified by centrifugation of the cell suspension on a 5 μm filter membrane (Millpore, Darmstadt, Germany) at 600× *g* for 5 min.

### Cell proliferation assay

The tachyzoites of *T. gondii* ME-49 were diluted to three different concentrations of 5 × 10^5^ cells/ml, 1 × 10^6^ cells/ml, and 5 × 10^6^ cells/ml, by 1640 medium, and added to 24-well plates containing Jurkat T-cells and Molt-4 T-cells (1 × 10^5^ cells/ml); the control group was treated with equal volume medium; each group had three accessory foramina, cultured for 48 h. At the end of the culture, 10 μl of CCK-8 solution (Dojindo, Kumamoto-ken Japan) was added into each well, and the cells were incubated at 37 °C for 2 h. Absorbance was read at 450 nm. Cell proliferation viability was calculated as follows:

$$ \mathrm{Proliferation}\ \mathrm{viability}\ \left(\%\right)=\left[\mathrm{A}\left(\mathrm{dosing}\right)\hbox{-} \mathrm{A}\ \left(\mathrm{blank}\right)\right]/\left[\mathrm{A}\ \left(\mathrm{control}\right)\hbox{-} \mathrm{A}\ \left(\mathrm{blank}\right)\right]\times 100 $$

where A (dosing) is the culture medium containing cells (CCK-8, substance to be measured); A (blank) is the culture medium containing cells (CCK-8, no substance to be measured); and A (control) is the culture medium without cells and substance to be measured, CCK-8.

### Cell apoptosis assay

Jurkat T-cells and Molt-4 T-cells were inoculated in 6-well plates and infected with tachyzoites (1 × 10^6^ cells/ml and 5 × 10^6^ cells/ml) for 48 h, respectively. After collection, cells were stained with the Annexin V-FITC and propidium iodide (PI) kit (KeyGen Biotech, Nanjing, China) in the dark for 5 min. Finally, cell apoptosis was quantified by flow cytometry on a Beckman Coulter flow cytometer.

### The Tunel assay

After the *T. gondii* ME-49 infection experiment was completed, the cells were collected by low-speed centrifugation and washed with PBS. The cells were fixed in 4% paraformaldehyde for 1 h. After the end of the fixation, cells were washed once with PBS. PBS was added to 0.1% Triton X-100 and incubated for 2 min in an ice bath. After the ice bath incubation, the treated cells were washed twice with PBS, 50 μl Tunel detection solution was added, and cells were incubated at 37 °C for 60 min. Finally, the smears were observed under a fluorescent microscope.

### MALT1 inhibitor

MI 2 (MALT1 inhibitor) is a small molecule and irreversible inhibitor of MALT1 with an IC_50_ of 5.84 μM. MI 2 MALT1 inhibitor was diluted with DMSO and added to Jurkat T-cells and Molt-4 T-cells for 24 h, the treatment time based on published MI 2 MALT1 inhibition studies [28].

### Western blot analysis

Jurkat T-cells and Molt-4 T-cells (2 × 10^6^ cells/ml) were cultured in 6-well plates and infected with tachyzoites at parasite: cell ratios of 1:4, 1:2 and 5:2 for 48 h. The cells were lysed in 100 μl lysis buffer containing 1% protease inhibitor. Protein concentration was determined with the colourimetric BCA assay. Proteins were separated by electrophoresis in a 10% SDS-polyacrylamide gel and transferred onto a PVDF membrane. The membrane was blocked with 5% (w/v) skimmed dry milk constituted in 1× TBST for 1.5 h at room temperature. Rabbit monoclonal anti-NF-κB p65 (1:1000), anti-phospho-NF-κB p65, anti-A20 (1:1000), anti-ABIN1 (1:1000), cleaved caspase-8 (1:1000) and mouse monoclonal anti-GAPDH (1:1000) antibodies were added overnight at 4 °C, and the respective horseradish peroxidase-conjugated secondary antibodies were then added as directed by the manufacturer for 1 h at room temperature. Immunoreactive bands were visualised using the enhanced chemiluminescence light (ECL) detection reagent.

### Lentiviral shRNA knock-down of A20 and ABIN1on Jurkat T-cells and Molt-4 T-cells

Lentiviruses bearing shRNA sequences specific to A20 and ABIN1 were purchased from Obio Co., Ltd. (Guangzhou, China). The target sequences were: (A20) 5'-CCC TCA TCG ACA GAA ACA T-3'; (ABIN1) 5'-AAC TCG CGC CTC TTC CAT CTG-3'. Jurkat T-cells and Molt-4 T-cells (1 × 10^6^ cells/ml) were centrifuged at 1000× *rpm* for 5 min to collect the precipitation of cells, and then the latter was diluted with 100 μl serum-free culture medium. 50 × 10^6^ lentivirus particles and 10 μg/ml polybrene were added to the cultured cells (MOI = 50), incubated at 30 °C, and centrifuged at 1200× *rpm* for 30 h. The treated cells were transferred to the culture plate, 1 ml 1640 medium was added to each culture plate; the medium was changed every 12 h. The efficiency of infection was confirmed by observation of immunofluorescence and Western blot analysis after 96 h.

### TNIP1-3Flag-IRES2-EGFP recombinant plasmid transfection

A plasmid expressing ABIN1 was constructed by Obio Co., Ltd. (China). The primers used were (forward: 5'-GAA CCG TCA GAT CCG CTA GCG CCA CCA TGG AAG GGA GAG-3' and reverse: 5'-ATT CGA AGC TTG AGC TCG AGC TGA GGC CCC TCA CG-3'.

DNA fragments were cloned into the Pum-T vector and transformed into DH5α cells. Positive clones were then selected for enzymatic identification and sequencing. A TNIP1 fragment digested with the *Nhe*I-HF and *Xho*I restriction enzymes was cloned into the flag-IRES2-EGFP vector for transfection. The sequence of the target fragments was detected with a homologous reading frame. The TNIP1-3 Flag-IRES2-EGFP recombinant plasmid was transfected using Lipofectamine RNAiMAX (RiboBio, Guangzhou, China) according to the manufacturer's instructions. The efficiency of transfection was confirmed by Western blot analysis after 72 h.

### Statistical analysis

All data are presented as the mean ± standard deviation (SD) and were analysed using SPSS software 24.0. Tests for homogeneity of variance were performed before analysis of variance. The differences between groups were analysed by Student’s t-test or one-way analysis of variance (ANOVA). *P* < 0.05 was considered as a statistically significant difference.

## Results

### *Toxoplasma gondii* ME-49 inhibits proliferation and induces apoptosis in human T-cell leukemia cell lines

**Fig. 1:**
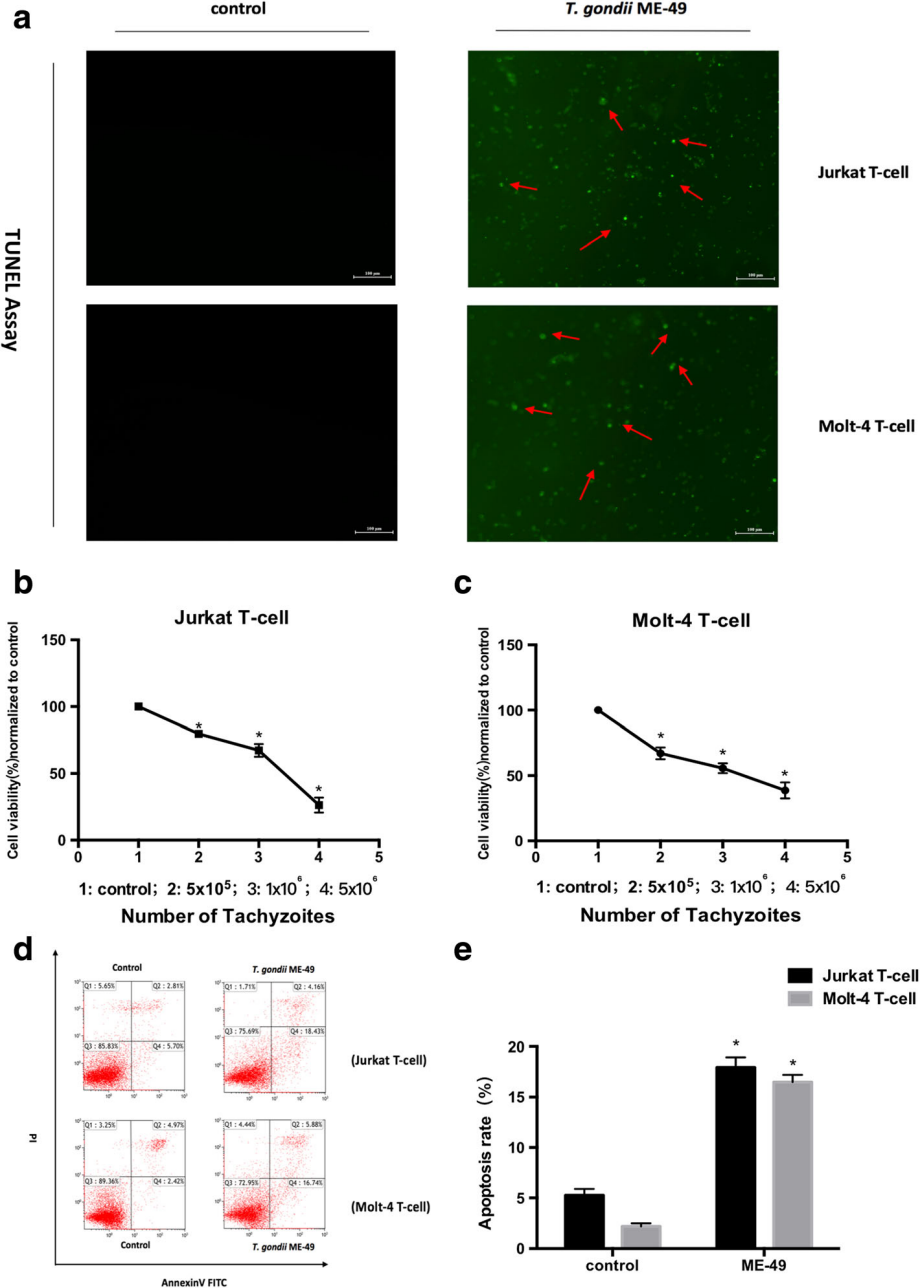
Effects of *T. gondii* ME-49 on Jurkat T-cell and Molt-4 T-cell proliferation and apoptosis in different concentration tachyzoites. **a** Tunel assay was used to detect the effect of *T. gondii* ME-49 on apoptosis of Jurkat T-cells and Molt-4 T-cells. **b, c** Effects of *T. gondii* ME-49 (5 × 10^6^ cells/ml) on Jurkat T-cell and Molt-4 T-cell proliferation. **d, e** Flow cytograms of cell apoptosis of cells infected with *T. gondii* ME-49 (5 × 10^6^ cells/ml). Q4, early apoptosis; Q2, late apoptosis. Values are presented as the mean ± SD (*n* = 3). *P < 0.05 *vs* the control group

To study the effect of *T. gondii* ME-49 on apoptosis of human T-cell leukaemia cell lines, we performed a Tunel assay on human leukaemia T-cells infected with *T. gondii* ME-49, and observed a large number of apoptotic cells with green fluorescence under a fluorescence microscope (Fig. 1a). In addition, we used different numbers of *T. gondii* tachyzoites to infect the human T-cell leukaemia cell lines Jurkat and Molt-4, and found the proliferation of these lines was inhibited by the CCK-8 assay (Jurkat T-cells *F*_(3, 8)_ = 199.74, *P <* 0.0001; Molt-4 T-cells *F*_(3, 8)_ = 110.59, *P <* 0.0001; Fig. 1b, c), and significant apoptosis was detected by flow cytometry assay (Jurkat T-cells *t*_(4)_ = -25.61, *P <* 0.0001; Molt-4 T-cells *t*_(4)_ = -44.82, *P <* 0.0001; Fig. 1d, e).

### *Toxoplasma gondii* ME-49 upregulate the level of A20 by inhibiting the TCR-mediated signalling pathway

**Fig. 2:**
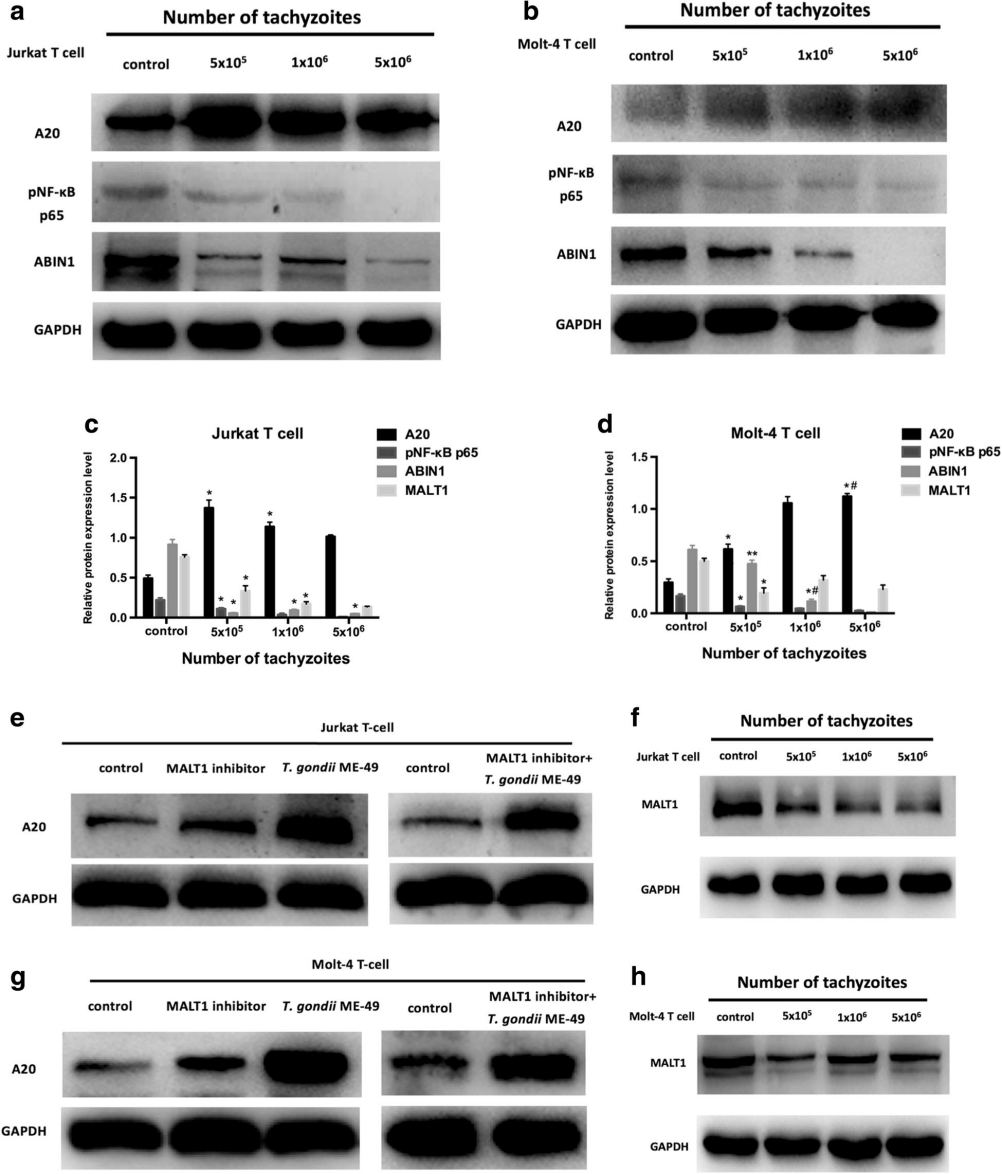
Effects of *T. gondii* ME-49 on A20, pNF-κB p65, ABIN1, MALT1 protein levels. **a, b, c, d** Effects of *T. gondii* ME-49 on A20, pNF-κB p65, ABIN1 protein levels in Jurkat T-cells and Molt-4 T-cells. **e, g** The A20 protein levels in Jurkat T-cells and Molt-4 T-cells treated with MALT1 inhibitor MI-2 and combined use of MI-2 and ME-49. **f, h** The MALT1 protein levels in Jurkat T-cells and Molt-4 T-cells infected with *T. gondii* ME-49 at parasite: cell rations of 1:4, 1:2, 5:2 for 48 h. Values are presented as the mean ± SD (*n* = 3). *P < 0.05 *vs* the control group; **P < 0.01 *vs* the control group; #*P* < 0.05 *vs* the 5 × 10^5^ cells/ml group

After we infected Jurkat T-cells with *T. gondii* ME-49, the level of A20 was upregulated (Jurkat T-cells *F*_(3, 8)_ = 212.38, *P* < 0.0001), ABIN1 downregulated (Jurkat T-cells *F*_(3, 8)_ = 957.78, *P* < 0.0001, Fig. 2a, c). Similar changes in A20 and ABIN1 levels also occurred in Molt-4 T-cells (A20: Molt-4 T-cells *F*_(3, 8)_ = 419.34, *P* < 0.0001; ABIN1: Molt-4 T-cells *F*_(3, 8)_ = 559.63, *P* < 0.0001, Fig. 2b, d). Previous studies have found that TCR stimulation can induce MALT1 paracaspase-mediated cleavage of A20, and continuous activation of NF-κB can also induce A20 expression. Here, we found the level of NF-κB p65 phosphorylation was significantly downregulated (Jurkat T-cells *F*_(3, 8)_ = 122.94, *P* < 0.0001; Molt-4 T-cells *F*_(3, 8)_ = 154.41, *P* < 0.0001), which demonstrated that A20 was not upregulated by inhibiting NF-κB activation. We found the MALT1 protein levels were decreased after *T. gondii* ME-49 infection (Jurkat T-cells *F*_(3, 8)_ = 330.41, *P* < 0.0001; Molt-4 T-cells *F*_(3, 8)_ = 65.49, *P* < 0.0001, Fig. 2f, h). In Jurkat T-cells and Molt-4 T-cells treated with MI-2 (MALT1 inhibitor), the levels of A20 were significantly upregulated (Fig. 2e, g). These data suggest that *T. gondii* ME-49 can upregulate the levels of A20 by inhibiting the TCR signalling pathway.

### A20 inhibits NF-κB activation and promotes apoptosis in human leukaemia T-cell lines

**Fig. 3:**
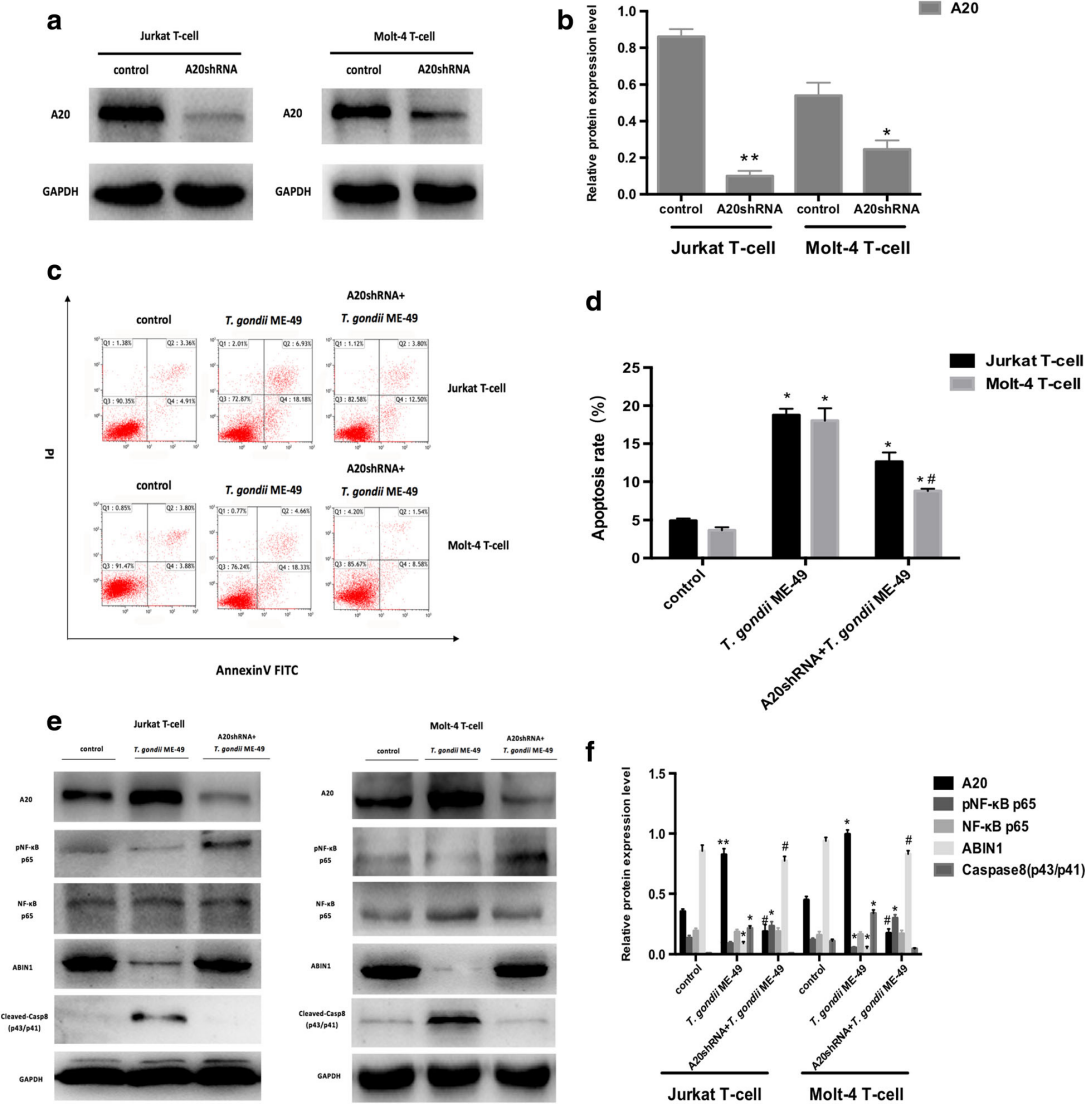
Effects of *T. gondii* ME-49 on A20, pNF-κB p65, NF-κB p65, ABIN1, Cleaved-Casp8 (p43/p41) expression levels and the apoptosis effects induced by *T. gondii* ME-49 after A20 knockdown. **a, b** Western blot analysis of knockdown efficiency of lentivirus in Jurkat T-cells and Molt-4 T-cells. **c, d** The apoptosis rate in Jurkat T-cells and Molt-4 T-cells infected with *T. gondii* ME-49 (5 × 10^6^ cells/ml) after A20 knockdown. **e, f** The A20, pNF-κB p65, NF-κB p65, ABIN1, Cleaved-Casp8 (p43/p41) protein levels in Jurkat T-cells and Molt-4 T-cells infected with *T. gondii* ME-49 (5 × 10^6^ cells/ml) after A20 knockdown. Values are presented as the mean ± SD (*n* = 3). *P < 0.05 *vs* the control group; **P < 0.01 *vs* the control group; #*P* < 0.05 *vs* the ME-49 group

To study the effect of A20 on apoptosis in human T-cell leukaemia cell lines, we constructed the lentivirus shRNA to knockdown the *A20* gene of Jurkat T-cells and Molt-4 T-cells, and found that the apoptosis rate of the two cell lines significantly decreased after *T. gondii* ME-49 infection (Jurkat T-cells *F*_(2, 6)_ = 190.928, *P* < 0.0001; Molt-4 T-cells *F*_(2, 6)_ = 167.23, *P* < 0.0001, Fig. 3c, d). By detecting the levels of NF-κB p65 phosphorylation, ABIN1, and cleaved Caspase-8, we determined the level of NF-κB p65 phosphorylation (Jurkat T-cells *F*_(2, 6)_ = 52.18, *P* < 0.0001; Molt-4 T-cells *F*_(2, 6)_ = 276.14, *P* < 0.0001). The ABIN1 protein level (Jurkat T-cells *F*_(2, 6)_ = 808.87, *P* < 0.0001; Molt-4 T-cells *F*_(2, 6)_ = 2354.33, *P* < 0.0001) were higher than in the non-knockdown group, and the levels of cleaved Caspase-8 were downregulated (Jurkat T-cells *F*_(2, 6)_ = 545.29, *P* < 0.0001; Molt-4 T-cells *F*_(2, 6)_ = 411.86, *P* < 0.0001, Fig. 3e, f).

### ABIN1 protect human leukemia T-cells by resisting the apoptosis induced by *T. gondii* ME-49

**Fig. 4:**
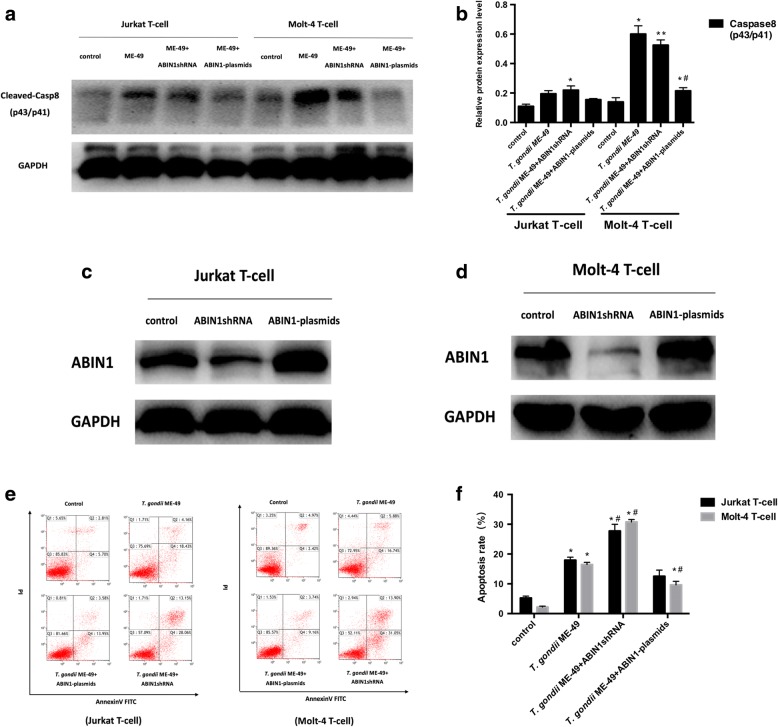
The apoptosis effects induced by *T. gondii* ME-49 after ABIN1 knockdown and over-expression of ABIN1 and effects of *T. gondii* ME-49 on the Cleaved-Casp8 (p43/p41) expression levels. **a, b** The Cleaved-Casp8 (p43/p41) protein levels in Jurkat T-cells and Molt-4 T-cells infected with *T. gondii* ME-49 (5 × 10^6^ cells/ml) after ABIN1 knockdown and over-expression of ABIN1. **c, d** Western blot analysis of knockdown efficiency of lentivirus and over-expression plasmid in Jurkat T-cells and Molt-4 T-cells. **e, f** The apoptosis rate in Jurkat T-cells and Molt-4 T-cells infected with *T. gondii* ME-49 (5 × 10^6^ cells/ml) after ABIN1 knockdown and over-expression of ABIN1. Values are represented as the mean ± SD (*n* = 3). *P < 0.05 *vs* the control group; **P < 0.01 *vs* the control group; #*P* < 0.05 *vs* the ME-49 group

Previous studies have shown that ABIN1 can have an anti-apoptotic effect by inhibiting the activation of Caspase-8. Here, the level of ABIN1 was upregulated, and the activation level of caspase-8 was significantly decreased by knockdown of A20 gene expression (Fig. 3e, f). To confirm that there was a regulatory relationship between the ABIN1 and caspase-8 proteins, we used different concentrations of tachyzoites to infect Jurkat T-cells and Molt-4 T-cells for which ABIN1 gene has been knocked- down, and found the level of cleaved Caspase-8 was upregulated (Jurkat T-cells: 0.22 ± 0.02) than the non-knockdown group (Jurkat T-cells: 0.20 ± 0.0, Fig. 4a, b), and the apoptosis rate of the two cell lines increased (Jurkat T-cells: 27.74 ± 1.57; Molt-4 T-cells: 30.75 ± 0.56) relative to the non-knockdown group (Jurkat T-cells: 17.89 ± 0.72; Molt-4 T-cells: 16.49 ± 0.51, Fig. 4e, f). Also, we used different concentrations of tachyzoites to infect Jurkat T-cells and Molt-4 T-cells for which ABIN1 gene has been overexpressed, and found that the apoptosis rate of Molt-4 T-cells decreased (Jurkat T-cells: 12.55 ± 1.45; Molt-4 T-cells: 9.63 ± 0.85) compared to the non-knockdown group (Jurkat T-cells: 17.89 ± 0.72; Molt-4 T-cells: 16.49 ± 0.51, Fig. 4e, f), and the cleaved Caspase-8 level was downregulated (Molt-4 T-cells: 0.22 ± 0.02, Fig. 4a, b) relative to the non-knockdown group (Molt-4 T-cells: 0.60 ± 0.04). Our data indicate that ABIN1 protects human leukaemia T-cells by resisting the apoptosis induced by *T. gondii* ME-49 *via* inhibition of the activation of caspase-8.

## Discussion

In this study, we confirmed that the *T. gondii* ME-49 strain could inhibit the proliferation of Jurkat T-cells and Molt-4 T-cells and promote apoptosis through the NF-κB signal pathway. The function of *T. gondii* ME-49 inhibiting proliferation and promoting apoptosis is related to upregulation of A20 expression.

Previous studies have shown that the expression of TNFR1 in mice infected with *T. gondii* is significantly increased, which is closely related to the immune regulation and apoptosis of host cells. Here, we examined the expression of nuclear factor NF-κB and related regulatory proteins downstream of TNFR1, and explained the mechanism of *T. gondii*-induced apoptosis in leukaemia T-cells *via* the NF-κB pathway. NF-κB is constitutively active in human cancer cells and is also a strong anti-apoptotic factor [29, 30]. Once NF-κB is activated, it will upregulate the expression of the downstream anti-apoptotic genes A20 and ABIN1, which suppress the excessive activation of NF-κB through a negative feedback mechanism [31, 32].

After using *T. gondii* ME-49 to infect the human leukaemia T-cell lines Jurkat and Molt-4, we found that the level of A20 was upregulated and the level of NF-κB p65 phosphorylation and ABIN1 was downregulated in the two cell lines. Mucosa-associated lymphoid tissue 1 (MALT1) is a key protein for TCR-induced T-cell activation [33, 34]. Previous studies have found that TCR stimulation can induce MALT1 paracaspase-mediated cleavage of A20 [35, 36]. Here, we detected a significant downregulation of MALT1 level, and found that under combined treatment with MI-2 and *T. gondii* ME-49, the level of A20 was significantly upregulated. These data suggest that *T. gondii* ME-49 can upregulate the level of A20 by inhibiting the TCR signalling pathway.

A20 is a ubiquitin chain enzyme that inhibits NF-κB activation in a negative feedback mechanism. Many studies have reported that A20 can have an indirect pro-apoptotic effect by inhibiting NF-κB activation [23]. To investigate the effect of A20 on the apoptosis induced by *T. gondii* ME-49, we used T. *gondii* ME-49 to infect the Jurkat T-cells and Molt-4 T-cells for which A20 gene has been knocked down, and found that the levels of NF-κB p65 phosphorylation and ABIN1 were significantly upregulated compared with the non-knockdown group cells, and that the apoptosis rate of two cell lines decreased. These results suggest that A20 can inhibit NF-κB activation and promote apoptosis in human leukaemia T-cells. Our research is similar to that of Kumar et al. [20], who showed NF-κB and related cytokines were significantly increased after knockdown of A20. Furthermore, we found that the level of 43/41-kDa cleaved caspase-8 (apoptosis-activated protein) was significantly downregulated. Since caspase-8 is the intermediate product of the death receptor pathway, our study also confirms that *T. gondii* can induce apoptosis *via* this pathway.

ABIN1 is the downstream target gene of NF-κB, which is widely expressed in lymphoid tissue cells [37]. Oshima et al. [26] found that under the induction of TNFα, a ABIN1 protein, can block the recruitment of FADD receptor to the caspase-8 and have an anti-apoptotic effect by directly binding to the ubiquitin chain. Previously, we found that *T. gondii* can downregulate the expression of ABIN1 in human leukaemia T-cells after *T. gondii* ME-49 infection. By constructing the lentiviral-mediated shRNA to knock down the ABIN1gene of Jurkat T-cells and Molt-4 T-cells and taking recombinant expression plasmid contained ABIN1 gene transfected into two cell lines, and then using *T. gondii* ME-49 to infect these lines, we found that the apoptosis rates increased and decreased respectively, and the level of 43/41-kDa cleaved Caspase-8 exhibited similar changes. These results suggest that ABIN1 can protect human leukaemia T-cells by helping to resist the apoptosis induced by *T. gondii* ME-49*,* and ABIN1 may be a potential negative regulator of death receptor-induced apoptosis. Previously, we found that the level of ABIN1 was upregulated after A20 gene knockdown in response to *T. gondii* ME-49 infection. This indicates that A20 can have an indirect pro-apoptotic effect by downregulating the level of ABIN1.

## Conclusions

Our data suggest that the *T. gondii* ME-49 strain can upregulate A20 expression and inhibit NF-κB activation by inhibiting the TCR signalling pathway. Additionally, ABIN1, an anti-apoptotic protein that negatively regulates NF-κB activity, was significantly downregulated in T-cells infected with *T. gondii* ME-49, suggesting that ABIN1 may not be involved in the inhibitory effect of A20 on NF-κB. However, after transfection of ABIN1 over-expression plasmid and after lentivirus knockdown of ABIN1, we found that the level of caspase-8 decreased and increased respectively, which indicated that ABIN1 could resist the apoptosis induced by *T. gondii* ME-49 by regulating the caspase-8 level. Our study provides a feasible mechanism for *T. gondii* to induce apoptosis in human leukaemia T-cells through the NF-κB pathway and related regulatory proteins.

## Abbreviations

DISC: death-inducing signalling complex
MALT1: mucosa-associated lymphoid tissue lymphoma translocation protein 1
NF-κB: nuclear factor kappa B
TCR: T-cell receptor
